# Potential Drug-Drug Interactions among Patients with Schizophrenia Spectrum Disorders: Prevalence, Association with Risk Factors, and Replicate Analysis in 2021

**DOI:** 10.3390/medicina59020284

**Published:** 2023-02-01

**Authors:** Cvetka Bačar Bole, Katja Nagode, Mitja Pišlar, Aleš Mrhar, Iztok Grabnar, Tomaž Vovk

**Affiliations:** 1 Psychiatric Hospital Idrija, 5280 Idrija, Slovenia; 2 Faculty of Pharmacy, University of Ljubljana, 1000 Ljubljana, Slovenia

**Keywords:** potential drug—drug interactions, schizophrenia, antipsychotics, drug interaction program, symptoms and signs, risk factors

## Abstract

*Background and Objectives*: Patients with schizophrenia are often exposed to polypharmacotherapy, which may lead to drug—drug interactions. The aim of the study was to investigate the prevalence of potential drug—drug interactions (pDDIs) in hospitalized patients with schizophrenia spectrum disorders and to identify factors associated with pDDIs and manifested symptoms and signs. *Materials and Methods*: This cross-sectional observational study included 311 inpatients admitted to a psychiatric hospital. The LexiComp drug interaction program was used to identify pDDIs in 2014. Factors associated with the prevalence of pDDIs and factors related to clinically observed symptoms and signs were assessed using multivariable regression. In addition, replicate analysis of pDDI was performed using 2021 program updates. *Results:* The prevalence of pDDIs was 88.7%. Our study showed that more than half of the patients received at least one drug combination that should be avoided. The most common pDDIs involved combinations of two antipsychotics or combinations of antipsychotics and benzodiazepines, which can lead to cardio-respiratory depression, sedation, arrhythmias, anticholinergic effects, and neuroleptic malignant syndrome. The number of prescribed drugs was a risk factor for pDDIs (OR 2.85; 95% CI 1.84–5.73). All groups of clinically observed symptoms and signs were associated with the number of drugs. In addition, symptoms and signs characteristic of the nervous system and psychiatric disorders were associated with antipsychotic dosage (IRR 1.33; 95% CI 1.12–1.58), which could contribute to the development of extrapyramidal syndrome, insomnia, anxiety, agitation, and bipolar mania. The 2021 version of the drug interaction program showed a shift in drug interactions toward a lower risk rating, implying less severe patient management and possibly less alert fatigue. *Conclusions:* Patients with schizophrenia spectrum disorders are at high risk of developing drug—drug interactions. Optimization of drug therapy, patient monitoring, and use of drug interaction programs could help to prevent pDDIs and subsequent adverse drug events.

## 1. Introduction

Schizophrenia, schizotypal, delusional, and schizoaffective disorders, shorter schizophrenia spectrum disorders (SSDs), are psychotic disorders classified in the International Classification of Diseases under codes F20-F29 [1]. SSDs typically include symptoms such as delusions, hallucinations, gross distortions of reality testing, disorganized speech, and behaviour that impair a person’s mental abilities, affective response, and ability to perceive reality and communicate [2]. The cornerstones of pharmacotherapy for patients with SSDs are antipsychotic drugs. Based on their mechanism of action, they can be divided into typical first-generation antipsychotics, which inhibit dopamine D2 receptors, and atypical second-generation antipsychotics, which inhibit dopaminergic and serotonergic receptors. Second generation antipsychotics are more commonly prescribed because of their high efficacy and fewer side effects. Extrapyramidal symptoms, neuroleptic malignant syndrome, and hyperprolactinemia are the main adverse effects of the first-generation antipsychotics. However, diabetes and metabolic adverse effects such as hyperlipidemia are more common with the second-generation antipsychotics [3]. Monotherapy with antipsychotics is the standard treatment recommended in many treatment guidelines [4,5]. However, because of the limited efficacy of antipsychotic monotherapy, antipsychotic polypharmacotherapy is commonly used, and the global median rate of antipsychotic polypharmacotherapy has been estimated to be approximately 20% [6], with substantial variation across geographic regions [7]. Patients with SSDs have a high prevalence of comorbidities including obesity, diabetes, hypertension, and dyslipidemia [8]. Because of polymorbidity and the resulting polypharmacotherapy, patients with SSDs are at high risk for developing drug—drug interactions (DDIs), which in turn further increase the likelihood of developing adverse drug events [9,10,11].

A DDI is a clinically significant change in the effect of a drug as a result of the concomitant administration of another drug and is usually considered to be either pharmacodynamic or pharmacokinetic [12]. Some DDIs may be beneficial and therefore used in therapy to optimize therapeutic effect. However, DDIs can also lead to adverse drug events and should be avoided [12]. A subcategory of DDI is potential DDI (pDDI), defined as an occurrence in which two drugs are known to interact when prescribed concomitantly, regardless of whether adverse events have occurred [12]. pDDIs should be distinguished from actual DDIs observed clinically in patients [13]. Many studies focusing on DDIs in psychiatric patients have been published and they are mainly addressing specific drug classes [14,15,16] or adverse reactions [17,18,19,20]. Most of the studies report pDDIs in psychiatric patients using retrospective data and various drug interaction database programs [10,20,21,22,23]. Beside the drug interaction programs, some authors have defined specific criteria to determine pDDIs in psychiatric patients that include a combination of drugs that are cytochrome inducers or inhibitors and corresponding sacrificial drugs, concomitant administration of more than one anticholinergic, and drugs that potentially prolong the QT interval [9,24]. The main limitation of studies addressing pDDIs in psychiatric patients is that they define pDDI based on different criteria. Consequently, numerous studies that compared different drug interaction database programs reported poor agreement [25,26,27]. Furthermore, there is poor literature evidence to support pDDIs, leaving uncertainty about the clinical relevance of pDDIs warnings in drug interaction programs [28,29,30,31]. Therefore, clinical studies addressing the association between pDDI and clinically observed adverse drug events are needed.

The present study is the follow-up analysis of the cohort of patients with SSDs described in our previous publications [32,33]. The patient cohort was first analyzed in 2014 with the aim of determining the prevalence of pDDIs, risk rating and the association of demographic characteristics and pharmacotherapy with pDDIs or clinically expressed symptoms and signs. In addition, we replicated the analysis of pDDIs in 2021 to examine the impact of drug interaction program updates on the pDDI risk rating and resulting treatment optimization recommendations.

## 2. Materials and Methods

### 2.1. Study Design and Population

Data were collected as part of a cross-sectional observational study at the Psychiatric hospital Idrija, Slovenia. We included all patients with SSDs, 18 to 65 years of age, who were hospitalized between December 2009 and March 2011. SSDs were diagnosed according to the International Classification of Diseases (codes F20-F29). The exclusion criteria were patients with liver and/or kidney disease, and pregnant women. No patient dropped out of the study. The study was approved by the National Medical Ethics Committee of the Republic of Slovenia (registration number: 112/11/19).

### 2.2. Data Source

Patients’ medical records were reviewed to obtain the following information: hospital admission and discharge, demographics, diagnoses, comorbidities, drug treatment, and patient symptoms and signs during hospitalization.

### 2.3. Potential Drug—Drug Interactions Dataset

pDDIs were identified using Lexicomp^®^ Online clinical decision support tool. The interaction module assigns pDDI risk ratings to the selected drugs: no known interaction (risk rating A), no action needed (risk rating B), monitor therapy (risk rating C), consider therapy modification (risk rating D), and avoid combination (risk rating X). In addition, the severity indicator describing the severity of the pDDI outcome is defined as minor, moderate, and severe, whereas the rating of the level of evidence of the pDDI is determined by the reliability rating as poor, moderate, good, and excellent [34]. pDDIs were first analyzed in 2014. We identified pDDIs among all concomitantly administered drugs in patients during a single hospitalization. After identifying pDDIs, patients were divided into two groups. Group 1 included patients with at least three type X pDDIs. Patients in group 1 could have additional pDDIs of a lower type. The mechanism and clinical manifestation of each type X and D pDDI were studied. Group 2 included patients who did not have type X and D pDDIs but could have type C, B, A, or no pDDIs. The remaining hospitalized patients were excluded from further analysis. Patient pharmacotherapy was reanalyzed in 2021 using the updated version of Lexicomp. The replicate analyses were performed with the same patients’ pharmacotherapy dataset that was used for identification of pDDIs in 2014. Polypharmacotherapy was defined as the prescription of five or more drugs.

In addition, symptoms and signs were identified in patients in groups 1 and 2 using three different strategies: (i) patients’ medical records were reviewed to identify symptoms and signs recorded during hospitalization; (ii) clinical pharmacists conducted interviews with psychiatrists and nurses; (iii) symptoms and signs were predicted based on drugs used to eliminate or alleviate side effects of antipsychotics, such as the use of biperiden to treat extrapyramidal symptoms caused by antipsychotics. The symptoms and signs were classified into five different groups: (i) nervous system and psychiatric disorders; (ii) gastrointestinal, hepatobiliary, metabolic, endocrine, renal, and urinary disorders; (iii) cardiovascular disorders; (iv) respiratory disorders; (v) other (see Supplementary). Patients in group 1 and 2 were compared to identify risk factors for pDDIs and risk factors for clinically observed symptoms and signs.

### 2.4. Statistical Analysis

Data are presented as medians, frequencies, or percentages. Comparisons were made using the Pearson Chi test for categorical independent and McNemar’s test for categorical paired variables and the Mann—Whitney U test for continuous variables. Logistic regression was performed to determine the association between the prevalence of pDDIs (patients in group 1 and 2) and age, sex, number of drugs, number of antipsychotics, and antipsychotic dosage (ratio of prescribed daily dose (PDD) of antipsychotic to maintenance daily dose (DDD) of antipsychotic for its main indication in adults, PDD/DDD). All independent variables were considered as continuous variables, except for sex (1 = male, 0 = female) and PDD/DDD (1 = PDD/DDD ≥ 1; 0 = PDD/DDD < 1). Univariate analysis was performed first, followed by multivariate analysis. Poisson regression was used to identify the risk factors in patients group 1 and 2 associated with the number of symptoms and signs (expressed as incident rate ratio—IRR). Exploratory variables included patient age, number of drugs, number of antipsychotics, and antipsychotic dosage (PDD/DDD) as continuous variables. In addition, patient gender and the occurrence of pDDIs (three or more type X pDDIs = 1, no type X and D pDDIs = 0) were considered as categorical variables. A *p* value of less than 0.05 was considered statistically significant. SPSS for Windows version 27, R software version 4.1.1, and RStudio version 2021.09.0 were used for statistical analysis.

## 3. Results

### 3.1. General Characteristics of the Patients

Table 1 shows the characteristics of the study population, which included 311 patients, the majority of whom were male (58%). The median age of the study population was 42 years, and the median time of hospitalization was 34 days. Patients received 2 antipsychotics and a total of 7 drugs. The comparison between group 1 and group 2 showed that there was no difference in gender and age of the patients. However, patients in group 1 were hospitalized longer (79 versus 31 days), received more drugs (12 versus 4) and antipsychotics (4 versus 2), were more often exposed to polypharmacotherapy (100% patients versus 37%), and had a higher dosage of antipsychotics (1.7 versus 0.93) than patients in group 2 (Table 1).

### 3.2. Prevalence of Potential Drug-Drug Interactions

**Figure 1:**
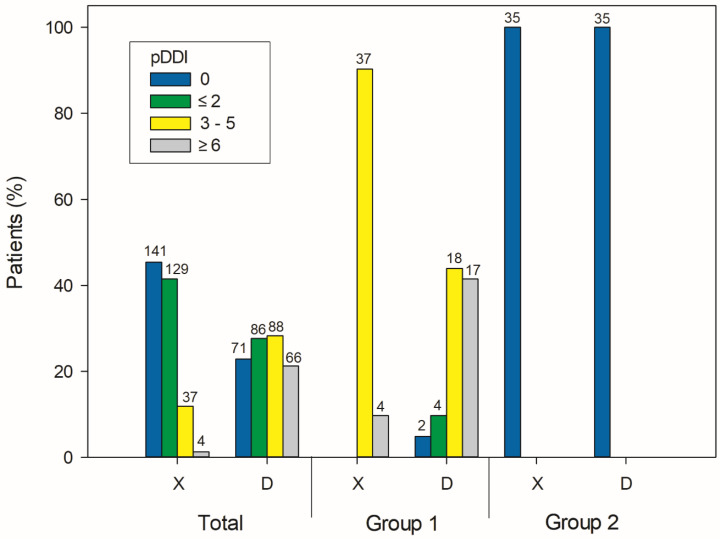
Prevalence of type X and D pDDIs. The total number of patients was 311, with 41 and 35 patients included in group 1 and group 2, respectively. The numbers above the bars indicate the number of patients.

Figure 1 illustrates the prevalence of type X and D pDDIs. There were 54.6% of patients who were exposed to at least one type X pDDI, and the majority of them were exposed to one or two type X interactions. In addition, 77.1% of patients were prescribed a drug combination that resulted in at least one type D pDDI. There were 41 patients who met the inclusion criteria for group 1, while group 2 included 35 patients. Furthermore, 90.2% of patients in group 1 were exposed to three to five type X pDDIs, and 85.3% were exposed to more than three type D pDDIs.

The 10 most commonly prescribed drug pairs leading to type X pDDI are listed in Table 2. The most commonly prescribed drug pairs include combinations of benzodiazepines and olanzapine. Olanzapine may potentiate the adverse or toxic effects of benzodiazepines, which manifest clinically as cardiorespiratory depression and excessive sedation. Combinations of two antipsychotics such as the combination of haloperidol and quetiapine or flupenthixol may prolong QT interval. The top 10 type X pDDIs also include combinations of two drugs with anticholinergic effects and the combination of amisulpride and clozapine, which can lead to the symptoms and signs characteristic of neuroleptic malignant syndrome. All 10 type X pDDIs can lead to major severity outcome, and nine out of 10 have good evidence, meaning they are documented in well-controlled studies.

### 3.3. Risk Factors for Type X Potential Drug—Drug Interactions

Table 3 shows the logistic regression analysis based on exposure to pDDIs for patients in group 1 and 2. In the univariate model, the association for pDDI was statistically significant with sex (OR = 0.37; *p* = 0.037), number of drugs (OR = 2.67; *p* < 0.001), number of antipsychotics (OR = 6.62; *p* < 0.001), and dosage of antipsychotics (OR = 4.85; *p* = 0.003). The multivariate model showed a significant association of pDDI only with the number of drugs (OR = 2.85; *p* < 0.001). However, the number and dosage of antipsychotics were excluded from the analysis due to collinearity with the number of drugs.

**Figure 2:**
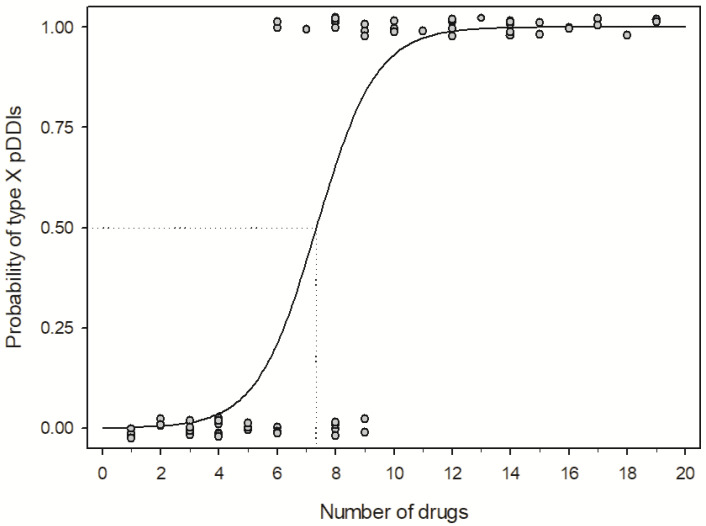
The probability of type X pDDIs in groups 1 and 2 of the studied population and association with the number of drugs.

The probability of type X pDDIs was compared between groups 1 and 2 of the studied population, and the association with the number of drugs that patients received is shown in Figure 2. The results showed that in patients taking 7 drugs simultaneously, the probability of at least three type X pDDIs was 50%.

### 3.4. Risk Factors for the Development of Clinically Observed Symptoms and Signs 

The risk factors for the development of clinically expressed symptoms and signs in patients group 1 and 2 were examined using Poisson regression (Table 4). Multivariate analyses showed that symptoms and signs characteristic of the nervous system and psychiatric disorders were significantly associated with number of drugs (IRR = 1.06; *p* < 0.001) and dosage of antipsychotics (IRR = 1.33; *p* = 0.012). Symptoms and signs characteristic of gastrointestinal, hepatobiliary, metabolic, endocrine, renal, and urinary disorders were significantly associated with sex (IRR = 0.66; *p* = 0.031) and number of drugs (IRR = 1.14; *p* < 0.001). The third group of symptoms and signs characteristic of cardiovascular disorders was significantly associated with the number of drugs (IRR = 1.22; *p* = 0.012), while the fourth group, which included symptoms and signs of respiratory disorders, was also significantly associated with the number of drugs (IRR = 1.19; *p* 0.043). The last group included all other clinically observed symptoms and signs and was significantly associated with number of drugs (IRR = 1.22; *p* < 0.001) and type X pDDIs (IRR = 0.28; *p* = 0.006). Thus, the number of drugs is associated with all groups of symptoms and signs, and each additional drug increases the risk for symptoms and signs by 6 to 22%.

### 3.5. Replicate Analysis of Potential Drug-Drug Interactions in 2021

To examine the impact of drug interaction program updates on pDDIs, we analysed the same cohort of inpatients using the Lexicomp^®^ online in 2014 and 2021. The results presented in Table 5 show that fewer patients had type X and D pDDIs when the 2021 database program was used. We also found a decrease in the total number of type X pDDIs and the number of different type X and D pDDIs when the 2021 results are compared with the 2014 results. Using the same criteria for classifying patients into groups 1 and 2, we demonstrated that the 2021 database program assigned fewer patients to group 1 and more patients to group 2 than the 2014 database program. Comparing the number of type X pDDIs identified in 2014 with the type X pDDIs identified in 2021, we found that only 38 of 78 type X pDDIs remained in the same category, with the remaining type X pDDIs having changed to type D or a lower DDI type. In addition, 16 new type X pDDIs were defined in 2021. A very similar pattern of changes in type D pDDIs is observed in the compared years. About half of the type D pDDIs identified in 2014 remained in the same category, while others changed to the lower type of pDDIs. Analysis of the most common type X pDDIs showed that combinations of olanzapine and benzodiazepines were the most common, regardless of the program selected. The combination of haloperidol and quetiapine, defined as a type X pDDIs in 2014, was changed to a type C pDDI, and the new combination of amisulpride and clozapine appeared in the top 5 type X pDDIs in 2021.

## 4. Discussion

Patients with SSDs are often exposed to polypharmacotherapy, which can lead to DDIs. DDIs pose a major therapeutic challenge because they can contribute to the occurrence of adverse drug events. It is known that adverse drug events can affect patient outcomes and pharmacotherapy efficacy and increase healthcare costs. In order to improve pharmacotherapy in patients with SSDs, studies focusing on DDIs are important.

The prevalence of pDDIs, assuming that type X and D pDDIs are clinically relevant [35,36,37], was 88.7%. The determined prevalence is high; however, it is comparable to studies focusing on psychiatric patients, as the determined prevalence ranged from 23 to 95% [10,21,23,38,39,40]. Castilho et al. reported that the prevalence of pDDI in hospitalized elderly patients diagnosed with a psychiatric disorder ranged from 67 to 81% depending on hospitalization stay [10]. Authors who studied psychiatric inpatients reported that the overall prevalence of pDDIs ranged from 65 to 77% [21,23,38]. Ocana-Zurita et al. studied outpatients diagnosed with schizophrenia and prevalence of pDDIs assuming the contribution of major and moderate pDDIs was 95% [40]. The lowest prevalence of pDDIs was 23% and was reported for adult schizophrenia patients with at least one prescribed antipsychotic [39]. The wide variation in the reported prevalence of pDDI is likely due to study design, population variability, study setting, drug interaction database program selection, and pDDI type criteria, making comparison difficult. Nevertheless, most studies report a prevalence of pDDI greater than 65% [10,21,23,38,40], confirming that psychiatric patients are at higher risk for DDI.

Our study showed that more than half of the patients were exposed to at least one drug combination that should be avoided (type X pDDI). Type X pDDIs most frequently involved antipsychotics, as all of the 10 most frequently observed drug pairs contained drugs with antipsychotic activity (Table 2). In addition to combinations of two antipsychotics, combinations of antipsychotics and benzodiazepines most frequently lead to type X pDDIs. Analysis of pDDIs for the groups of antiparkinsonic, antiepileptic, and antidepressant drugs showed that only the combinations with antidepressants lead to type X pDDIs. The identified type X pDDIs may cause cardiorespiratory depression and excessive sedation when combinations of benzodiazepines and olanzapine are used. On the other hand, combinations of two antipsychotics may lead to prolongation of the QT interval or excessive anticholinergic activity. It has been reported that pDDIs between olanzapine and benzodiazepines are very common in psychiatric patients. Using Lexicomp, Rancovic et al. reported that such pDDIs were observed in 5.3 to 9.4% of psychiatric patients, depending on the specific benzodiazepine used [22], which is comparable to our observation. Aburamadan et al. also found that 3.1% of psychiatric patients were taking diazepam and olanzapine, which may lead to pDDI [38]. The combination of haloperidol with quetiapine or flupenthixol may lead to a prolongation of the QT interval, which was the second most common manifestation of pDDIs in our study. In some studies, reporting pDDIs in psychiatric patients, an even more pronounced occurrence of increased risk for prolonged QT was observed, and such a consequence was found in 5 to 7 of the 10 most common pDDIs [10,38]. The potential for a prolonged QT interval was more pronounced with first-generation antipsychotics than with those of the second generation, and parenteral application of haloperidol had the highest risk [41]. In addition, the risk for prolongation of the QT interval increases with polypharmacotherapy and involves combinations of antipsychotics and antidepressants [42,43]. The last potential clinical consequence of the most common pDDIs was the anticholinergic effect. This finding is consistent with the study by Ocana-Zurita et al. and Ismail et al. [21,40], but other studies analyzing pDDIs in psychiatric patients did not report such interactions.

To further investigate pDDIs, patients were divided into two groups representing the extremes of the studied population. The first group included patients with more than three type X pDDIs and the second group included those who did not have type X and D pDDIs. We found a strong association of pDDIs with the increasing number of drugs, which is consistent with other studies in psychiatric patients [10,21,22,38]. In addition, patients receiving 7 drugs had a 50% probability of having at least three type X pDDIs. However, we found no association with age, sex, and number or dosage of antipsychotic drugs, which is not consistent with other studies [10,22,39]. Dosage and number of antipsychotics corelate with number of drugs, therefore we were not able to differ between them. The lack of association with age was probably due to the relatively young population (median: 42 years) with a narrow age interval (1st and 3rd interquartile range: 31–52 years). The most frequent comorbidities in group 1 and group 2 were type 2 diabetes, arterial hypertension, hypokalaemia, hypothyroidism, osteoporosis, and other mental and cognitive disorders. 

The advantage of the present study is that we associated clinically observed symptoms and signs with demographic characteristics and pharmacotherapy of psychiatric patients, because such analyses are rarely performed. The dominant variable associated with all groups of symptoms and signs was the number of drugs that patients received. This is not surprising, since observed symptoms and signs required pharmacological treatment and thus increased the number of drugs that patients received. The most common symptoms and signs characteristic of the nervous system and psychiatric disorders in patient group 1 were extrapyramidal syndrome, insomnia, anxiety, agitation, and bipolar mania (see Supplementary). These symptoms and signs could be related to an inadequate effect of antipsychotics, which explains the confirmed association with antipsychotic dosage (Table 4). This group of symptoms and signs was also associated with the number of drugs and type X pDDIs (slightly above statistical significance), suggesting a possible contribution of pDDIs to their development. The most common symptoms and signs characteristic of gastrointestinal, hepatobiliary, metabolic, endocrine, renal, and urinary disorders were constipation, weight loss, stomach pain, weight gain, and hypercholesterolemia. It was interesting that the symptoms and signs characteristic of the respiratory system (obstructive bronchitis, asthma, chronic obstructive pulmonary disease, caught, pulmonary embolism) occurred only in the patients with more than 3 type X pDDI (group 1) and not in the patients without type X or D pDDI (group 2). In addition to number of drugs, smoking could also contribute to the development of respiratory disorder, as more than 70% of patients were smokers. Among other symptoms and signs that were frequently observed in the patients group 1 were pain, fever, and bacterial infections. These symptoms and signs are characteristic of infections. Many studies have confirmed that patients with schizophrenia are at increased risk for developing infections [44,45,46] and pneumonia represents one of the major causes for their death [46]. A retrospective analysis of Japanese patients with schizophrenia found that advanced age, low body mass index, smoking, and use of antipsychotics and their high dosage were risk factors for pneumonia [46]. Increased antipsychotic dosage was just above statistical significance among tested variables, thus showing a potential association with symptoms and signs of group Others. In contrast, we found out that type X pDDIs represent decreased risk for development of symptoms and signs of the group Others.

The final objective of the present study was to determine the influence of drug interaction program updates on the assigned pDDI category by replicating the analysis for the same cohort of patients using the program in 2021. Our analyses clearly show that when the newer version of the drug interaction database program was used, the number of type X pDDIs decreased and that the majority of the former type X pDDIs were moved to category C or even to a lower category. Consequently, the number of patients with type X pDDI decreased significantly. In addition, the newer version of the drug interaction database program showed that the number of patients with type D pDDIs has also decreased. A similar pattern of changes in type D pDDIs was observed as in type X pDDIs, since part of type D pDDIs remained in the same category and all other changes were to a lower category with no changes to type X pDDIs. These results apparently indicate that the drug—drug interactions studied were classified in a lower risk rating, implying that less severe patient management would be required. The risk rating for a specific drug—drug interaction is determined based on data sources that include clinical data from electronic health records, insurance claims, clinical trial data, post-marketing surveillance systems, scientific literature, etc. Advances in the field of DDIs are contributing to a re-evaluation of pDDI categories with the goal of obtaining systems that would better support clinical decisions. Based on our results, we can speculate that a smaller number of clinically important pDDIs will reduce the number of alerts, thereby reducing alert fatigue, which has been reported to be one of the major problems of drug interaction database programs [47,48,49]. Monteith and Glenn compared 100 drug interaction pairs, including psychiatric drugs, in six different drug interaction database programs in 2018 and 2020, and confirmed that the database programs were inconsistent in both years studied and there were no clear upward or downward trends of rating changes over time, which is contrary to our findings [50]. We believe that the different study period may have contributed to the results. The results of pharmacotherapy analysis using the drug interactions program database clearly indicate that additional measures are needed to improve pharmacotherapy in psychiatric patients, independent of program updates. One potential solution is to integrate clinical pharmacist services into regular clinical practice. Positive effects of clinical pharmacist interventions such as reductions in the number of prescribed drugs, potentially inappropriate medications, and type X pDDIs have been described in the literature [51].

The complexity of the data analyzed warns to interpret the results with caution and to consider the limitations of the study. The main limitation of our study is that the data were collected in a single psychiatric hospital, so it may be difficult to generalize the results. The study was non-interventional and therefore pDDIs were not resolved. We used only a single drug interaction program to determine pDDIs, thus the results are not directly applicable to other drug interaction programs. Finally, the causality of the observed symptoms and signs was not investigated, therefore we were unable to adequately account for the clinical manifestation of DDIs. Nevertheless, we demonstrated associations between clinically observed symptoms and signs and several variables, including pDDIs, indicating at least a possible association.

## 5. Conclusions

In the present study, a high prevalence for pDDIs was found in hospitalized patients with schizophrenia spectrum disorders. Patients receiving a larger number of drugs and combinations of two antipsychotics or combinations of antipsychotics and benzodiazepines are at high risk for developing drug—drug interactions. We have been able to associate symptoms and signs characteristic of nervous system and psychiatric disorders with the number of drugs and dosage of antipsychotics. Therefore, close monitoring of patients and optimization of pharmacotherapy are recommended to control and prevent the clinical consequences of drug—drug interactions. The drug interaction database programs could be a valuable drug interaction management tool and could be implemented to clinical practice via clinical pharmacist services. However, the large number of alerts and some uncertainty about the clinical relevance of pDDIs make clinical decisions difficult, with high probability of ignoring alerts even those with high clinical relevance. The replicated analysis using the updated drug interaction program showed some progress, yet major improvements are still needed to better support clinical decisions.

## Figures and Tables

**Table 1:** Characteristics of patients.

	Total No. (%)	Group 1 ^a^	Group 2 ^b^
Patients, n	311 (100)	41 (13)	35 (11)
Men, n	179 (58)	17 (42)	23 (66)
Women, n	132 (43)	24 (59)	12 (34)
Age [years], median (Q_1_–Q_3_)	42 (31–52)	44 (32–52)	43 (27–51)
Smokers, n	213 (68)	30 (73)	28 (80)
Time of hospitalization [days], median (Q_1_–Q_3_)	34 (18–69)	79 (50–142)	31.0 (16–61) *
Number of hospitalizations, median (Q_1_–Q_3_)	1 (1–2)	1 (1–2)	1 (1–1) *
Number of prescribed drugs, median (Q_1_–Q_3_)	7 (5–10)	12 (8–15)	4 (2–6) *
Polypharmacotherapy, n (%) ^c^	242 (77.8)	41 (100)	13 (37.1) *
Number of antipsychotics, median (Q_1_–Q_3_)	2 (2–3)	4 (3–5)	2 (1–2) *
Antipsychotic PDD/DDD, median (Q_1_–Q_3_)	/	1.7 (1.3–2.1)	0.93 (0.73–1.50) *

^a^—at least 3 type X pDDIs; ^b^—no interactions of type X or D pDDIs; ^c^—polypharmacotherapy means number of patients that received at least five drugs; n—number of patients; Q_1_—first quartile (25th percentile); Q_3_—third quartile (75th percentile); *—*p* < 0.05, between group 1 and 2; PDD/DDD—ratio between prescribed daily dose (PDD) of antipsychotic versus maintenance dose per day (DDD) of antipsychotic drug for its main indication in adults; number of drugs, number of antipsychotics drugs and antipsychotic PDD/DDD are defined per hospitalization; /—not available.

**Table 2:** Ten most common type X pDDIs in all patients (n = 311), the summary of interaction, patient management, severity, and reliability rating.

Type X Potential Drug—Drug Interaction		No. of Patients (%)	Summary	Patient Management	Interaction Severity	Reliability Rating
Diazepam	Olanzapine	59 (19.0)	Olanzapine may enhance the adverse/toxic effect of benzodiazepines.	Additive adverse effects: cardiorespiratory depression, excessive sedation.	major	good
Lorazepam	Olanzapine	26 (8.4)				
Alprazolam	Olanzapine	23 (7.4)				
Midazolam	Olanzapine	12 (3.9)				
Haloperidol	Quetiapine	24 (7.7)	QT-prolonging antipsychotics.	Monitor for QTc interval prolongation and ventricular arrhythmias.	major	good
Haloperidol	Flupenthixol	9 (2.9)				
Clozapine	Flupenthixol	12 (3.9)	Anticholinergic agents may enhance the adverse/toxic effect of other anticholinergic agents.	Additive anticholinergic effects: dry mouth, dry eyes, blurred vision, urinary retention, and constipation.	major	good
Clozapine	Quetiapine	10 (3.2)			major	good
Quetiapine	Zuclopenthixol	9 (2.9)			major	good
Amisulpride	Clozapine	9 (2.9)	Amisulpride may enhance the adverse/toxic effect of antipsychotics.	Symptoms and signs of neuroleptic malignant syndrome.	major	fair

**Table 3:** Logistic regression analysis based on exposure to potential drug—drug interactions (n = 76).

Variables	Univariate Model		Multivariate Model	
	OR (95% CI)	*p*-Value	OR (95% CI)	*P*-Value
Age	1.02 (0.98–1.06)	0.293	0.97 (0.89–1.04)	0.376
Sex	**0.37 (0.14–0.93)**	**0.037**	0.48 (0.07–2.81)	0.425
Number of drugs	**2.67 (1.79–5.02)**	**<0.001**	**2.85 (1.84–5.73)**	**<0.001**
Number of antipsychotics	**6.62 (3.19–18.04)**	**<0.001**	/ ^#^	/ ^#^
PDD/DDD	**4.85 (1.75–14.73)**	**0.003**	/ ^#^	/ ^#^

Dependant variable was presence of 3 or more type X pDDI (group 1) versus no clinically relevant pDDI (group 2); continues variables are age, number of drugs, and number of antipsychotics; dichotomous variables are sex (1 = male, 0 = female), and PDD/DDD (1 = PDD/DDD ≥ 1; 0 = PDD/DDD < 1); ^#^ collinearity between number of drugs and number and dosage of antipsychotics; OR—odds ratio; CI—confidence interval; PDD/DDD—ratio between prescribed daily dose (PDD) of antipsychotic versus maintenance dose per day (DDD) of antipsychotic drug for its main indication in adults.

**Table 4:** Poisson regression analysis based on exposure to clinically observed symptoms and signs for patients in group 1 and 2 (n = 76).

Symptoms and Signs	Multivariate Model	Variables				
		Age	Sex	Number of Drugs	PDD/DDD	Type X pDDIs
Nervous system and psychiatric disorders	IRR (95% CI)	1.00 (0.99–1.01)	1.09 (0.86–1.39)	**1.06** **(1.04–1.09)**	**1.33** **(1.12–1.58)**	1.46 (0.99–2.17)
	*p*-value	0.913	0.499	**<0.001**	**0.012**	0.099
Gastrointestinal, hepatobiliary, metabolic, endocrine, renal and urinary disorders	IRR (95% CI)	1.00 (0.99–1.01)	**0.66** **(0.45–0.97)**	**1.14** **(1.09–1.19)**	1.10 (0.84–1.43)	0.81 (0.48–1.38)
	*p*-value	0.799	**0.031**	**<0.001**	0.540	0.562
Cardiac and vascular disorders	IRR (95% CI)	1.03 (0.99–1.08)	0.50 (0.18–1.36)	**1.22** **(1.02–1.45)**	0.73 (0.40–1.35)	0.55 (0.09–3.48)
	*p*-value	0.116	0.212	**0.012**	0.429	0.523
Respiratory disorders	IRR (95% CI)	1.06 (1.01–1.09)	0.67 (0.16–2.75)	**1.19** **(1.04–1.37)**	1.26 (0.55–2.89)	/
	*p*-value	0.095	0.530	**<0.043**	0.647	/
Others	IRR (95% CI) *p*-value	1.00 (0.98–1.03) 0.705	0.78 (0.47–1.31) 0.335	**1.22** **(1.15–1.30)** **<0.001**	1.42 (1.02–1.97) 0.102	**0.28** **(0.12–0.61)** **0.006**

Dependant variable was number of symptoms and signs; continues variables are age, number of drugs, and PDD/DDD; dichotomous variables are sex (1 = male, 0 = female) and type X pDDIs (1 = at least 3 type X pDDIs; 0 = no type X or D pDDIs); IRR—incidence rate ratios; CI—confidence interval; PDD/DDD—ratio between prescribed daily dose (PDD) of antipsychotic versus maintenance dose per day (DDD) of antipsychotic drug for its main indication in adults. Variables significantly associated with symptoms and signs are in bold.

**Table 5:** Comparison of pDDIs determined using the Lexicomp® drug interaction database program in 2014 and 2021.

	Analysis 2014	Analysis 2021
**pDDIs in all patients**		
Patients with type X pDDIs	170 **	121
Patients with type D pDDIs	240 **	207
Total number of type X pDDIs	308	217
Total number of type D pDDIs	942	1043
Number of different type X pDDIs	78	54
Number of different type D pDDIs	166	158
Max number of type X pDDIs per patient (type D)	10 (21)	9 (24)
**pDDIs in specific group**		
Group 1 ^a^	41	17
Group 2 ^b^	35	63
**Changes of pDDI in all patients**		
X → X	/	38
X → D	/	5
X → C, B, A, N	/	35
New X	/	16
D → D	/	75
D → X	/	0
D → C, B, A, N	/	91
New D	/	83
**Most frequent pDDIs**		
1. type X pDDIs (n)	Diazepam—Olanzapine (59)	Diazepam—Olanzapine (59)
2. type X pDDIs (n)	Lorazepam—Olanzapine (26)	Lorazepam—Olanzapine (26)
3. type X pDDIs (n)	Alprazolam—Olanzapine (23)	Alprazolam—Olanzapine (23)
4. type X pDDIs (n)	Haloperidol—Quetiapine (24)	Midazolam—Olanzapine (12)
5. type X pDDIs (n)	Midazolam—Olanzapine (12)	Amisulpride—Clozapine (9)

^a^ group 1—at least 3 type X pDDIs; ^b^ group 2—no type X or D pDDIs; **—statistical comparison between analysis in 2014 and 2021 (*p* < 0.001); type of pDDI according to Lexicomp^®^ Online: X, D, C, B, A; N—no interactions of Risk Level A or greater identified.

## Data Availability

The authors agree to make data and materials supporting the results or analyses presented in this paper available upon reasonable request.

## Supplementary Materials

The following supporting information can be downloaded at: https://www.mdpi.com/article/10.3390/medicina59020284/s1, Table S1. Symptoms and signs characteristic for patients with more than 3 type X pDDIs (patients group 1) and patients with no type X or D pDDIs (patients group 2).

## References

[1] International Statistical Classification of Diseases and Related Health Problems 10th Revision. https://icd.who.int/browse10/2010/en.

[2] Stahl S.M. (2008). The Prescriber’s Guide: Stahl’s Essential Psychopharmacology: Antipsychotics and Mood Stabilizers.

[3] Lakshmikuttyamma A., Haghparast P., Hajjar E., Smith T., Pooresfehani P., Ray S.D. (2020). Chapter 7—Antipsychotic agents. Side Effects of Drugs Annual.

[4] Hasan A., Falkai P., Wobrock T., Lieberman J., Glenthoj B., Gattaz W.F., Thibaut F., Moller H.J., WFSBP Task Force on Treatment Guidelines for Schizophrenia (2012). World Federation of Societies of Biological Psychiatry (WFSBP) Guidelines for Biological Treatment of Schizophrenia, part 1: Update 2012 on the acute treatment of schizophrenia and the management of treatment resistance. World J. Biol. Psychiatry.

[5] Barnes T.R., Drake R., Paton C., Cooper S.J., Deakin B., Ferrier I.N., Gregory C.J., Haddad P.M., Howes O.D., Jones I. (2020). Evidence-based guidelines for the pharmacological treatment of schizophrenia: Updated recommendations from the British Association for Psychopharmacology. J. Psychopharmacol..

[6] Gallego J.A., Bonetti J., Zhang J., Kane J.M., Correll C.U. (2012). Prevalence and correlates of antipsychotic polypharmacy: A systematic review and meta-regression of global and regional trends from the 1970s to 2009. Schizophr. Res..

[7] Kim J.J., Pae C.U., Han C., Bahk W.M., Lee S.J., Patkar A.A., Masand P.S. (2021). Exploring Hidden Issues in the Use of Antipsychotic Polypharmacy in the Treatment of Schizophrenia. Clin. Psychopharmacol. Neurosci..

[8] Viron M.J., Stern T.A. (2010). The impact of serious mental illness on health and healthcare. Psychosomatics.

[9] Wolff J., Hefner G., Normann C., Kaier K., Binder H., Hiemke C., Toto S., Domschke K., Marschollek M., Klimke A. (2021). Polypharmacy and the risk of drug-drug interactions and potentially inappropriate medications in hospital psychiatry. Pharmacoepidemiol. Drug Saf..

[10] Castilho E.C.D., Reis A.M.M., Borges T.L., Siqueira L.D.C., Miasso A.I. (2018). Potential drug-drug interactions and polypharmacy in institutionalized elderly patients in a public hospital in Brazil. J. Psychiatr. Ment. Health Nurs..

[11] English B.A., Dortch M., Ereshefsky L., Jhee S. (2012). Clinically significant psychotropic drug-drug interactions in the primary care setting. Curr. Psychiatry Rep..

[12] Hines L.E., Murphy J.E. (2011). Potentially harmful drug-drug interactions in the elderly: A review. Am. J. Geriatr. Pharmacother..

[13] Mallet L., Spinewine A., Huang A. (2007). The challenge of managing drug interactions in elderly people. Lancet.

[14] Buzea C.A., Dima L., Correll C.U., Manu P. (2022). Drug-drug interactions involving antipsychotics and antihypertensives. Expert Opin. Drug Metab. Toxicol..

[15] Goodlet K.J., Zmarlicka M.T., Peckham A.M. (2019). Drug-drug interactions and clinical considerations with co-administration of antiretrovirals and psychotropic drugs. CNS Spectr..

[16] Low Y., Setia S., Lima G. (2018). Drug-drug interactions involving antidepressants: Focus on desvenlafaxine. Neuropsychiatr. Dis. Treat..

[17] Toto S., Hefner G., Hahn M., Hiemke C., Roll S.C., Wolff J., Klimke A. (2021). Current use of anticholinergic medications in a large naturalistic sample of psychiatric patients. J. Neural Transm..

[18] Hefner G., Hahn M., Hiemke C., Toto S., Wolff J., Roll S.C., Klimke A. (2021). Pharmacodynamic Drug-Drug interactions of QT-prolonging drugs in hospitalized psychiatric patients. J. Neural Transm..

[19] Das B., Rawat V.S., Ramasubbu S.K., Kumar B. (2019). Frequency, characteristics and nature of risk factors associated with use of QT interval prolonging medications and related drug-drug interactions in a cohort of psychiatry patients. Therapie.

[20] Khan Q., Ismail M., Haider I., Khan F. (2017). Prevalence of QT interval prolonging drug-drug interactions (QT-DDIs) in psychiatry wards of tertiary care hospitals in Pakistan: A multicenter cross-sectional study. Int. J. Clin. Pharm..

[21] Ismail M., Iqbal Z., Khattak M.B., Javaid A., Khan M.I., Khan T.M., Asim S.M. (2012). Potential Drug-Drug Interactions in Psychiatric Ward of a Tertiary Care Hospital: Prevalence, Levels and Association with Risk Factors. Trop. J. Pharm. Res..

[22] Rankovic A., Milentijevic I., Jankovic S. (2022). Factors associated with potential drug-drug interactions in psychiatric inpatients. Eur. J. Hosp. Pharm..

[23] Kirilochev O.O., Dorfman I.P., Umerova A.R., Bataeva S.E. (2019). Potential drug-drug interactions in the psychiatric hospital: Frequency analysis. Res. Results Pharmacol..

[24] Wolff J., Hefner G., Normann C., Kaier K., Binder H., Domschke K., Hiemke C., Marschollek M., Klimke A. (2021). Predicting the risk of drug-drug interactions in psychiatric hospitals: A retrospective longitudinal pharmacovigilance study. BMJ Open.

[25] Muhic N., Mrhar A., Brvar M. (2017). Comparative analysis of three drug-drug interaction screening systems against probable clinically relevant drug-drug interactions: A prospective cohort study. Eur. J. Clin. Pharmacol..

[26] Liu X., Hatton R.C., Zhu Y., Hincapie-Castillo J.M., Bussing R., Barnicoat M., Winterstein A.G. (2017). Consistency of psychotropic drug-drug interactions listed in drug monographs. J. Am. Pharm. Assoc..

[27] Monteith S., Glenn T., Gitlin M., Bauer M. (2020). Potential Drug interactions with Drugs used for Bipolar Disorder: A Comparison of 6 Drug Interaction Database Programs. Pharmacopsychiatry.

[28] Nguyen T., Liu X., Abuhashem W., Bussing R., Winterstein A.G. (2020). Quality of Evidence Supporting Major Psychotropic Drug-Drug Interaction Warnings: A Systematic Literature Review. Pharmacotherapy.

[29] Magro L., Moretti U., Leone R. (2012). Epidemiology and characteristics of adverse drug reactions caused by drug-drug interactions. Expert Opin. Drug Saf..

[30] Paterno M.D., Maviglia S.M., Gorman P.N., Seger D.L., Yoshida E., Seger A.C., Bates D.W., Gandhi T.K. (2009). Tiering drug-drug interaction alerts by severity increases compliance rates. J. Am. Med. Inform. Assoc..

[31] Smithburger P.L., Buckley M.S., Bejian S., Burenheide K., Kane-Gill S.L. (2011). A critical evaluation of clinical decision support for the detection of drug-drug interactions. Expert Opin. Drug Saf..

[32] Bačar Bole C., Pislar M., Mrhar A., Tavcar R. (2017). Prescribing patterns for inpatients with schizophrenia spectrum disorders in a psychiatric hospital in Slovenia: Results of 16-month prospective, non-interventional clinical research. Psychiatr. Danub..

[33] Bačar Bole C., Pislar M., Sen M., Tavcar R., Mrhar A. (2017). Switching antipsychotics: Results of 16-month non-interventional, prospective, observational clinical research of inpatients with schizophrenia spectrum disorders. Acta Pharm..

[34] Lexicomp Online Lexicomp Drug Interactions Analysis. https://www.wolterskluwer.com/en/solutions/lexicomp/resources/lexicomp-user-academy/drug-interactions-analysis..

[35] Jazbar J., Locatelli I., Horvat N., Kos M. (2018). Clinically relevant potential drug-drug interactions among outpatients: A nationwide database study. Res. Social Adm. Pharm..

[36] Vegh A., Lanko E., Fittler A., Vida R.G., Miseta I., Takacs G., Botz L. (2014). Identification and evaluation of drug-supplement interactions in Hungarian hospital patients. Int. J. Clin. Pharm..

[37] Namazi S., Pourhatami S., Borhani-Haghighi A., Roosta S. (2014). Incidence of Potential Drug-Drug Interaction and Related Factors in Hospitalized Neurological Patients in two Iranian Teaching Hospitals. Iran. J. Med. Sci..

[38] Aburamadan H.A.R., Sridhar S.B., Tadross T.M. (2021). Assessment of potential drug interactions among psychiatric inpatients receiving antipsychotic therapy of a secondary care hospital, United Arab Emirates. J. Adv. Pharm. Technol. Res..

[39] Guo J.J., Wu J., Kelton C.M., Jing Y., Fan H., Keck P.E., Patel N.C. (2012). Exposure to potentially dangerous drug-drug interactions involving antipsychotics. Psychiatr. Serv..

[40] Ocana-Zurita M.C., Juarez-Rojop I.E., Genis A., Tovilla-Zarate C.A., Gonzalez-Castro T.B., Lilia Lopez-Narvaez M., de la O de la O M.E., Nicolini H. (2016). Potential drug-drug interaction in Mexican patients with schizophrenia. Int. J. Psychiatry Clin. Pract..

[41] Ozeki Y., Fujii K., Kurimoto N., Yamada N., Okawa M., Aoki T., Takahashi J., Ishida N., Horie M., Kunugi H. (2010). QTc prolongation and antipsychotic medications in a sample of 1017 patients with schizophrenia. Prog. Neuropsychopharmacol. Biol. Psychiatry.

[42] Ramasubbu S.K., Mishra A., Mandal S. (2022). Prevalence of QT-Prolonging Drug-Drug Interactions in Psychiatry: A Systematic Review and Meta Analysis. J. Pharm. Pract..

[43] Nose M., Bighelli I., Castellazzi M., Martinotti G., Carra G., Lucii C., Ostuzzi G., Sozzi F., Barbui C., Star Network G. (2016). Prevalence and correlates of QTc prolongation in Italian psychiatric care: Cross-sectional multicentre study. Epidemiol. Psychiatr. Sci..

[44] Han J., Shen M., Wan Q., Lv Z., Xiao L., Wang G. (2022). Risk factors for community-acquired pneumonia among inpatients with mental disorders in a tertiary general hospital. Front. Psychiatry.

[45] Pankiewicz-Dulacz M., Stenager E., Chen M., Stenager E.N. (2019). Risk factors of major infections in schizophrenia. A nationwide Danish register study. J. Psychosom. Res..

[46] Haga T., Ito K., Sakashita K., Iguchi M., Ono M., Tatsumi K. (2018). Risk factors for pneumonia in patients with schizophrenia. Neuropsychopharmacol. Rep..

[47] Bryant A.D., Fletcher G.S., Payne T.H. (2014). Drug interaction alert override rates in the Meaningful Use era: No evidence of progress. Appl. Clin. Inform..

[48] Isaac T., Weissman J.S., Davis R.B., Massagli M., Cyrulik A., Sands D.Z., Weingart S.N. (2009). Overrides of medication alerts in ambulatory care. Arch. Intern. Med..

[49] Poly T.N., Islam M.M., Yang H.C., Li Y.J. (2020). Appropriateness of Overridden Alerts in Computerized Physician Order Entry: Systematic Review. JMIR Med. Inform..

[50] Monteith S., Glenn T. (2021). Comparison of potential psychiatric drug interactions in six drug interaction database programs: A replication study after 2 years of updates. Hum. Psychopharmacol..

[51] Stuhec M., Lah L. (2021). Clinical pharmacist interventions in elderly patients with mental disorders in primary care focused on psychotropics: A retrospective pre-post observational study. Ther. Adv. Psychopharmacol..

