# Supplementary material for: Potential Drug-Drug Interactions among Patients with Schizophrenia Spectrum Disorders: Prevalence, Association with Risk Factors, and Replicate Analysis in 2021
Source: Medicina (Kaunas). 2023 Feb 1;59(2):284. doi: 10.3390/medicina59020284 (PMC9962414; doi:10.3390/medicina59020284)
Supplement: Supplementary file 1 [file medicina-59-00284-s001.zip › medicina-2119978-SI.pdf]

Supplementary, Table S1. Symptoms and signs characteristic for patients with more than 3 type X pDDIs (patients group 1) and patients with no type X or D pDDIs (patients group 2).

**Symptoms and signs**		
	Patients group 1[a]<br><br>(number/patient)	Patients group 2[b]<br><br>(number/patient)
Nervous system and psychiatric disorders	extrapyramidal symptoms (0.81)<br>insomnia (0.79)<br>anxiety (0.74)<br>agitation (0.71)<br>bipolar mania (0.49)<br>delusions/psychosis (0.27)<br>sedation (0.25)<br>anger/irritation (0.13)<br>headache (0.13)<br>depression (0.05)<br>vertigo (0.05)<br>crying (0.05)<br>oculogyric crisis (0.05)<br>lightheadness (0.05)<br>salivation (0.03)<br>elements of OCD (0.03)<br>epilepsy and neuropathic pain (0.03)<br>loss of consciousness (0.03)<br><br>Total symptoms/signs[c, **] = 0.09 (0.05 – 0.43)	extrapyramidal symptoms (0.43)<br>anxiety (0.34)<br>agitation (0.29)<br>bipolar mania (0.23)<br>insomnia (0.11)<br>headache (0.06)<br>delusions/psychosis (0.06)<br>epilepsy (0.03)<br>abnormal EEG (0.03)<br>sedation (0.03)<br>forgetfulness (0.03)<br>paranoia (0.03)<br><br>Total symptoms/signs[c] = 0.06 (0.03 – 0.25)
Gastrointestinal, hepatobiliary, metabolic, endocrine, renal, and urinary disorders	constipation (0.56)<br>weight loss (0.39)<br>pain in abdomen (0.34)<br>weight gain (0.27)<br>hypercholesterolemia (0.15)<br>stomach pain (0.12)<br>hyperglycaemia (0.12)<br>vomiting (0.05)<br>nausea (0.02)<br>abnormal liver function tests (0.02)<br>hypothyroidism (0.02)<br>galactorrhoea (0.02)<br>deficiency of vitamin C (0.02)<br>deficiency of vitamin B-complex (0.02)<br>urinary retention (0.02)<br><br>Total symptoms/signs[c, **] = 0.05 (0.02 – 0.21)	weight gain (0.31)<br>constipation (0.14)<br>weight loss (0.11)<br>pain in abdomen (0.06)<br>hypothyroidism (0.06)<br>stomach pain (0.06)<br>deficiency of vitamin B-complex (0.03)<br>reduced activity of the adrenal gland (0.03)<br>hyperglycaemia (0.03)<br>exocrine pancreatic insufficiency (0.03)<br><br>Total symptoms/signs[c] = 0.06 (0.03 – 0.10)
Cardiac and vascular disorders	essential hypertension (0.17)<br>tachycardia (0.12)<br>hypotension (0.05)	essential hypertension (0.06)<br>hypotension (0.039)<br>tachycardia (0.03)

	hypertension and tachycardia (0.02)<br><br>Total symptoms/signs[c] = 0.09 (0.04 – 0.13)	 <br>Total symptoms/signs[c] = 0.03 (0.03 – 0.04)
Respiratory disorders	obstructive bronchitis, asthma, COPD (0.15)<br>cough (0.07)<br>pulmonary embolism (0.07)<br>pneumonia (0.02)<br><br>Total symptoms/signs[c] = 0.07 (0.06 – 0.09)	/<br><br><br><br><br><br>Total symptoms/signs[c] = 0
Others	pain, fever (0.49)<br>bacterial infection (0.34)<br>hypokalemia (0.12)<br>toothache (0.1)<br>red eyes (0.02)<br>leukopenia (0.02)<br>neutropenia (0.02)<br>thrombocytopenia (0.02)<br>allergy (0.02)<br>anemia (0.02)<br>hyponatremia (0.02)<br>muscular hypotonia (0.02)<br>oedema of legs (0.02)<br><br>Total symptoms/signs[c, *] = 0.02 (0.02 – 0.10)	pain, fever (0.23)<br>bacterial infection (0.17)<br>hypokalemia (0.09)<br>toothache (0.03)<br>anemia (0.03)<br>allergy (0.03)<br>oedema of the upper and lower lip (0.03)<br>osteoporosis (0.03)<br><br>Total symptoms/signs[c] = 0.03 (0.03 – 0.11)

COPD – chronic obstructive pulmonary disease; EEG – electroencephalogram; OCD – obsessive compulsive disorder; [a] group 1 – at least 3 type X pDDIs; [b] group 2 – no type X or D pDDIs; [c] median (interquartile range 25th – 75th percentile); *, ** – comparison between symptoms and signs in patients group 1 and 2 (* $p < 0.05$; ** $p < 0.001$)
